# Evolution of post-deployment indicators of oral health on the Family Health Strategy

**DOI:** 10.1590/S1679-45082014AO3000

**Published:** 2014

**Authors:** Danielle da Costa Palacio, Fabiana de Lima Vazquez, Danielle Viana Ribeiro Ramos, Stela Verzinhasse Peres, Antonio Carlos Pereira, Luciane Miranda Guerra, Karine Laura Cortellazzi, Jaqueline Vilela Bulgareli

**Affiliations:** 1 Sociedade Beneficente Israelita Brasileira Albert Einstein, São Paulo, SP, Brazil.; 2 Faculdade de Odontologia de Piracicaba, Piracicaba, SP, Brazil.; 3 Universidade de São Paulo, São Paulo, SP, Brazil.

**Keywords:** Oral health, Family health, Absenteeism, Health services accessibility, Delivery of healthcare

## Abstract

**Objective:**

To evaluate the evolution of indicators after the implementation of 21 Oral Healthcare Teams in the Family Health Strategy.

**Methods:**

We used data from outpatient services of Oral Healthcare Teams to evaluate efficiency, access, percentage of absences and emergencies of oral healthcare professionals who worked in the partnership between the *Sociedade Beneficente Israelita Brasileira Hospital Albert Einstein *and the *Secretaria Municipal de Saúde de São Paulo*, during the period 2009-2011.

**Results:**

Percentages of emergencies, income, and access showed a significant difference during the period analyzed, but no difference for percentage of absences was found. When monthly analysis was made, it is noteworthy that at the beginning of service implementation a fluctuation occurred, which may indicate that the work was consolidated over the months, becoming capable of receiving new professionals and increasing the population served. Comparison of the indicators in that period with the goals agreed upon between the *Sociedade Beneficente Israelita Brasileira Hospital Albert Einstein* and the *Secretaria Municipal de Saúde de São Paulo* made it possible to notice that the Oral Health Teams had a good performance.

**Conclusion:**

The results showed that the goals were achieved reflecting the increasing number of professionals, the maturing of work processes in the Oral Health Teams, and optimization of the manpower available to perform the activities. Understanding these results will be important to guide the actions of Oral Health Teams for the following years and to assess the achievement of goals.

## INTRODUCTION

Actions of the Family Health Strategy have transformed the system of healthcare in Brazil and have been increasingly consolidated as an important instrument in promoting well-being of the population, with improved health and quality of life. Throughout this transformation, the Primary Care Units [UBS - *Unidade Básica de Saúde*] have been able to form multi-professional teams composed at minimum, by a physician, a nurse, one or two nursing assistants, and four to six community agents. Thus, the challenge of working within a multidisciplinary team appeared, with responsibility over a territory when a certain number of families live. This strategy brought with it the possibility of reclaiming the bonds of commitment and of co-responsibility among healthcare services, professionals, and the population. Therefore, one of the basic principles of this model of care is that of educating for health, going beyond a merely curative medical assistance.^(1)-3)^

For Baldani et al.,^(4)^ the fact of dentistry not being present in the Strategy from the beginning possibly caused losses in the process of integrating the various healthcare professionals, and may have determined various forms in the process of implementation of the Oral Healthcare Teams (OHTs).

According to Emmi and Barroso,^(5)^ to incorporate oral healthcare into the Family Health Strategy does not necessarily include the dentist (D), oral healthcare assistant (OHA), and the oral healthcare technician (OHTn) in the minimal team made up of physician, nurse, and nurse’s aide, but it requires mixing the work of these professionals with this OHT. Transdisciplinarity should occur for an efficient and resolutive progression of the work. The work process in oral health should be compatible and permeable to the work process of the other professional categories that work in the UBSs, in order to meet most of the demands of this assisted community, based on the bond, on the collective, and on the participation of this population.

If the Family Healthcare Teams (FHTs) and OHTs function adequately, they are able to resolve 85% of the health problems of their community with a good level of care, preventing disease, avoiding unnecessary hospitalization, and improving the quality of life of the population.^(6, 7)^

Despite the fact that the Family Health Strategy is presented as a restructuring model, we see that there still is a reproduction of traditional methods.^(7)^ In the implementation of the FHTs and OHTs, it is necessary to offer special attention to the qualification of the professionals, seeking integral care of the families, and not only changing the site of work, but especially, their conduct in face of the problems to be taken on. ^(8, 9)^

According to Athayde and Rodrigues,^(10)^ although much is said about the importance of team work, this practice faces various challenges to be overcome by all its components. One of them is the recognition of the diversity of knowledge and skills among members of the team, who should complement each other in order to enrich the work as a whole. In the work process in health, team work and the production of new practices in health are interdependent issues tied into a web in which it is difficult to define which is cause and which is consequence.

To Farias and Moura et al.,^(11)^ to recognize that there are concrete obstacles capable of hindering the fulfillment of the Family Health Strategy objectives is to acknowledge that there are challenges to be faced.

This considered, this project had the challenge of studying the exchanges of the OHTs of the partnership between the *Sociedade Beneficente Israelita Brasileira Albert Einstein *(SBIBAE) and the *Secretaria Municipal de Saúde de São Paulo* (SMS/SP), within this context of Family Health Strategy and in the territory of multiple social and population nuances.

## OBJECTIVE

To evaluate the progression of the indicators of efficiency, access, percentage of absences, and percentage of emergencies after implementation of the Oral Healthcare Teams in the Family Healthcare Strategy.

## METHODS

In the municipality of São Paulo, the FHTs began in 1996 through the state program called “Integral Quality in Health Project” [Projeto QUALIS - Qualidade Integral em Saúde].^(12)^ This program established partnerships with for-no-profit organizations that incorporate support assistance resources for the FHTs, such as the OHTs, mental health, and specialties ambulatories, even before oral health officially became a part of the Family Health Strategy.^(13, 14)^

The partnership between SBIBAE and SMS/SP established in 2001, initially only covered the FHTs in the region of Campo Limpo/Vila Andrade, which is in the southern zone of the city of São Paulo (SP). This region has a population with complexities equivalent to that of a medium sized city (more than 100 thousand inhabitants), with various social problems and many contrasts, with regions of low-income population agglomerations and residential complexes, besides middle- and high-class horizontal communities.

Although the term “Primary Care Unit” is not normally used in the national territory to treat units with FHTs, here this is how it was adopted, since it is the term used in the Municipality of São Paulo. In November 2008, oral health became a part of this partnership, with the installation of 12 OHTs modality I (1 D and 1 OHA), implanted in 6 UBSs.

Analyses of the variables of this study initiated in January of 2009. In April, 2009, a modality I OHTs was added (total: 13 OHTs in 6 UBSs). Still in 2009, in the month of October, 5 more OHTs were established and this enlargement reached 8 UBSs (total: 18 OHTs in 8 UBSs). Another important factor was that 14 OHTs of modality I (of the 18 existing OHTs) changed to modality II (when incorporated into OHTs). In March 2011, there was inclusion of one more UBS and the entry of one more 1 OHT modality I and 2 OHTs modality II (total: 21 OHTS in 9 UBSs).

The work process at OHTs was based on the constant directives of the guiding document of the *Prefeitura Municipal de São Paulo* – version 2009,^(15)^ which establishes various parameters, among which, 40 weekly hours of work, 30 of them directed towards clinical care, 2 hours to home visits, 2 hours to meetings, 2 hours to groups, and 4 hours to collective actions in schools of the area covered. Every week 49 patients should be scheduled for OHTs modality I and 56 patients for OHTs modality II, as well as emergencies in spontaneous demand. Additionally, 49 and 56 new patients (first dental appointment) should be seen monthly for OHTs modalities I and II, respectively, besides the percentage of emergencies and absences lower than 20%.

The study was submitted to and approved by the Ethics in Research Committee of the *Faculdade de Odontologia de Piracicaba da Universidade Estadual de Campinas* (FOP-UNICAMP), with protocol # 036/2013, according to the requirement of Resolution 196/96 on research involving human beings, registered by means of *Plataforma Brasil*.

The sample included the UBSs where OHTs participated in the partnership of SBIBAE and SMS/SP in the Campo Limpo/Vila Andrade region, including the entire population registered during the period of 2009 to 2011 in the Brazil’s Basic Healthcare Database *[SIAB - Sistema de Informação da Atenção].*

Data condensed from ambulatory production maps of the professionals at the OHTs was used and evaluated by the following items: total number of procedures carried out by the D, OHTn, and OHA; number of patients scheduled; number of emergency appointments; number of absences at scheduled appointments; number of consultations performed (scheduled patients + emergency patients – patients who missed their appointments); number of new cases per year (first dental consultation); and total number of tooth brushing events supervised in collective actions (dental brushing with the purpose of prevention, guided by an oral healthcare professional in a school environment which had been previously registered).

The following indicators were then calculated: percentage of absences (proportion of absences of the total number of appointments scheduled), percentage of emergencies (proportion of emergencies in the total number of appointments), efficiency (number of procedures performed/number in the total of patients treated), and access (number of new cases/number of the assigned population).

The descriptive analysis of the data was made by absolute and relative frequencies, medians, minimum and maximum values. The normality test was used to verify compliance of the variables analyzed with the normal curve using the Kolmogorov-Smirnov test.

Since the variables did not show a normal distribution, free distribution tests were used. To verify monthly evolution of the indicators percentage of absences, percentage of emergencies, and efficiency, Spearman’s statistical non-parametric correlation test was used. The difference between the years analyzed for the indicator of access and the other items cited above was measured by Kruskal-Wallis’s non-parametric test. For the post hoc analyses, Dunn’s test was used.

A 5% level was adopted for statistical significance. The data were entered into an Excel spreadsheet and analyzed with the Statistical Package for the Social Sciences (SPSS) version 20.0 for Windows and GraphPad InStat programs.

## RESULTS

According to SIAB, the population registered in the areas covered by the OHTs included populations with 136,749, 167,594, and 182,801 inhabitants, for the years 2009, 2010, and 2011, respectively, taking into consideration the number of UBSs with OHTs for each of the years cited. This population was formed, in the most part, by women (52.8%). We point out that the most predominant age range for all periods was 20 to 39 years (38.3%), followed by 40 to 49 years (12.9%).

Table 1 shows the sum of OHTs activities carried out during the years 2009, 2010, and 2011. Table 2 shows the growth percentage from 2009 to 2010, from 2010 to 2011, and from 2009 to 2011, of the variables that were presented on table 1. For a better understanding, both tables 1 and 2 are analyzed together. We highlight the increase in mean number of professionals active during the total period studied (78.12% growth), in which in 2010 relative to 2009, this increase was 56.25%, and in 2011 relative to 2010, was only 14%. From 2009 to 2011, there was an increase of 128.09% in the total number of procedures carried out by the professionals who were a part of the OHTs. In fact, in 2010, we noted an 87.35% increase relative to 2009, while in 2011 relative to 2010, the increase was more modest (21.72%). For the total number of patients seen (scheduled patients + emergency patients – patients who missed scheduled appointments), a 91.74% increase was noted from 2009 to 2011. The growth observed was more expressive in 2010 (50.28%) relative o 2009, and considerably smaller in 2011 (27.58%) relative to 2010.

**Table 1:** Absolute number of variables analyzed after implementation of the Oral Healthcare Teams in the region of Campo Limpo/Vila Andrade in São Paulo, during the years 2009, 2010, and 2011

Variable	Year		
	2009	2010	2011
Mean of active professionals (D + OHTn + OHA)	32	50	57
Total number of procedures (D + OHTn + OHA)	94,215	176,515	214,896
Total number of patients seen ([SP+EP]-MP)	19,499	29,305	37,389
SP	18,675	30,710	39,457
EP	4,878	6,249	7,204
MP	4,054	7,654	9,272
Patients at the first dental appointment	7,588	10,870	16,383
Supervised tooth brushing as a collective action	7,309	41,569	70,724

D: dentist; OHTn: oral healthcare technician; OHA: oral healthcare assistant; SP: scheduled patients; EP: emergency patients; MP: patients who missed scheduled appointments.

**Table 2:** Percentage comparison among the years analyzed on table 1

Variable	Growth (%)		
	2009-2010	2010-2011	2009-2011
Mean of active professionals (D+OHTn+OHA)	56.25	14	78.12
Total number of procedures (D+OHTn+OHA)	87.35	21.72	128.09
Total number of patients seen ([SP+EP]-MP)	50.28	27.58	91.74
SP	64.44	28.54	111.28
EP	28.10	15.28	47.68
MP	88.80	21.13	128.71
Patients at the first dental appointment	43.25	50.71	115.51
Supervised tooth brushing as a collective action	468.73	70.13	867.62

D: dentist; OHTn: oral healthcare technician; OHA: oral healthcare assistant; SP: scheduled patients; EP: emergency patients; MP: patients who missed scheduled appointments.

As to the fist dental appointments, a 115.51% increase was noted from 2009 to 2011. The difference from 2009 to 2010 and from 2010 to 2011 was not expressive (43.25% and 50.71%, respectively). Finally, a significant progress was noted in the number of supervised tooth brushings in school children, since the increase was 867.62% from 2009 to 2011. This increase was considerably greater during the period of 2010 relative to 2009, when the growth was 468.73%, than in 2011 relative to 2010, when the growth was 70.13%. The data were collected monthly and added to reach the total in reference to each year. The total number of procedures incorporated the data for all participants of each category of D, OHTn, and OHA.

Table 3 shows the annual results obtained as to the indicators access, efficiency, percentage of absences and emergencies, also year to year, for the 2009-2011 period. We noted that there was a significant difference between the years analyzed for the indicators percentage of emergencies (p=0.002), efficiency (p=0.009), and access (p<0.001). For the indicator percentage of absences, there was no statistically significant difference. In 2010, the smallest amplitude was recorded between the minimum and maximum of the indicator percentage of absences between the years studied. The same behavior was seen for the indicator percentage of emergencies, which in 2010 displayed the smallest disparity between the minimum and maximum between the years analyzed. We should point out a clear tendency of decline between the years 2009 (median of 26.86) and 2011 (median of 19.5). The difference between the minimum and maximum efficiency presented in 2009 was the most disparate among the years studied. The indicator of access was evaluated in an accumulated form by years, that is, for this variable, a sum was made month by month to reach the annual indicator. The result for the year 2011 was the most expressive in the period analyzed, with a clear tendency for improvement in the indicator. We chose the calculation of the median, and not of the mean due to the disparate difference between minimum and maximum of the indicators studied.

**Table 3:** Descriptive analysis of the indicators percentage of absences, emergencies, efficiency, and access for the 2009-2011 period

Variable	2009			2010			2011			p value*
	Median	Minimum	Maximum	Median	Minimum	Maximum	Median	Minimum	Maximum	
Percentage of absence	22.68	9.3	29.5	25.29	20.1	30.1	23.94	16.5	27.9	0.169
Percentage of emergencies	26.86	14.6	36.1	21.52	17.5	24.9	19.5	15.1	22.9	0.002
Efficiency	5.28	2.27	6.21	5.94	4.87	7.14	5.77	4.85	6.81	0.009
Access* *	5.5			6.5			9.0			<0.001

* Kruskal-Wallis; ** The access indicator was evaluated in an accumulated way per year; in this variable the total value is specified. Minimum and maximum refer to the smallest and greatest results shown between the months studied during the 2009-2011 period, respectively.

On table 4, we compare the results of the indicators: percentage of absences, emergencies, and efficiency for the 2009-2011 period, by means of Spearman’s correlation analysis. We noted no statistically significant correlation for the indicator percentage of absences in the period. As to percentage of emergencies, we noted a significant negative correlation. We highlight that the indicator efficiency showed a significant positive correlation (r=0.40; p=0.016).

**Table 4:** Analysis between the months of January 2009 and December 2011 for the indicators percentage of absences, emergencies, and efficiency

Variable	R	p value*
Percentage of absences	0.18	0.285
Percentage of emergencies	-0.58	<0.001
Mean monthly efficiency	0.40	0.016

* Spearman’s correlation.

**Figure 1:**
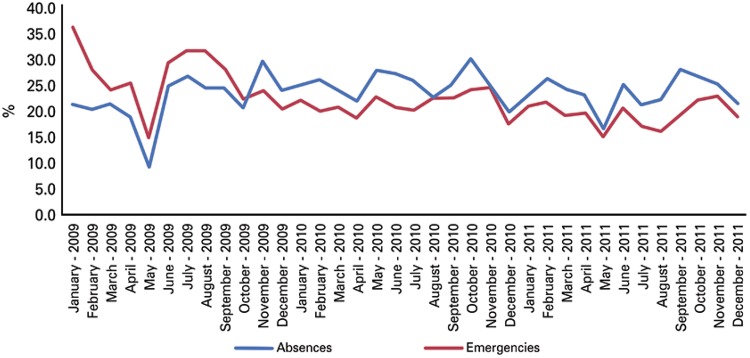
Evolution in percentage of absences and emergencies throughout the months during the period of 2009-2011

To better understand the behavior of the indicators percentage of absences, emergencies, and mean efficiency in a way that made sense of the correlation data found on table 3, we investigated the monthly evolution of these indicators. The indicator percentage of absences demonstrated an inconsistent behavior during the first months post-implementation of the OHTs, with adjustments of the less expressive variations as of June 2009 (Figure 1). The same inconsistent behavior was noted in the first months post-implementation for the indicator percentage of emergencies. The variations observed for this indicator, among the months throughout the study, were less expressive, but the pattern of general distribution showed a slight tendency to decline between the initial and final collection timepoints. We observed an abrupt reduction of both indicators in the month of May 2009, and in the case of percentage of emergencies, the drop was even more expressive, especially taking into consideration the initial data collected for this indicator.

**Figure 2:**
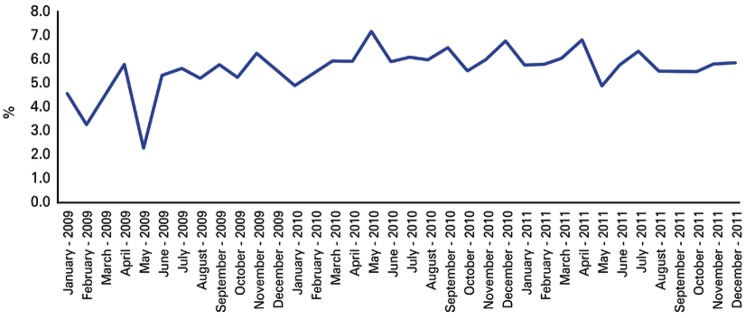
Evolution of mean efficiency throughout the months during the period of 2009-2011

The efficiency indicator (Figure 2) demonstrated a behavior similar to that of the indicators in figure 1,* i.e.*, being inconsistent in the first OHT post-implementation months and adjusting the less expressive variations as of June 2009. The efficiency indicator also showed less expressive variations in the following months of the study, with a general distribution pattern denoting a slight tendency of increasing between the initial and final collection timepoints. The month of May 2009 also demonstrated an abrupt reduction of this indicator.

Table 5 highlights that there was a statistically significant difference between the years analyzed for the indicators percentage of emergencies (p=0.002), mean monthly efficiency (p=0.009), and access (p<0.001). After the analysis of differences between the years, the post hoc analysis was performed to verify if the statistical significance was maintained for all the years. We noted a statistically significant difference between the years 2009 and 2010. As to mean monthly efficiency, the difference was observed between the years 2009 and 2011. As to access, the difference was noted for all the comparisons.

**Table 5:** Post hoc for multiple comparisons

Comparisons	p value*
Percentage of emergencies	
2009 *versus* 2010	<0.01
2009 *versus* 2011	>0.05
2010 *versus* 2011	>0.05
Efficiency	
2009 *versus* 2010	>0.05
2009 *versus* 2011	<0.01
2010 *versus* 2011	>0.05
Access	
2009 *versus* 2010	<0.01
2009 *versus* 2011	<0.01
2010 *versus* 2011	<0.01

* Dunn’s tests.

## DISCUSSION

In this study, we presented the evolution of the post-implementation results of the 21 OHTs in the partnership between SBIBAE and SMS/SP, during the period from 2009 to 2011, for an estimated general population of 264,697 inhabitants, considering the last year of the study. Comprehension of these results is important in order to guide the action of oral health at the OHTs for the following years, and to evaluate the fulfillment of goals and challenges launched by SMS/SP.

Among the few articles already pushed^(13)^,^14, 16)^, this is the first to involve the SMS/SP partnerships and other institutions, and it verified the evolution of the indicators access, efficiency, percentage of absences and emergencies of the OHTs, after its insertion into the Family Health Strategy. The data available in reference to the partnerships established by SMS/SP, or those published by municipalities of other states, focused on the process of implementation, on covering of the areas with this service, or on the satisfaction of the user, without the use of the indicators presented here.^(5, 6, 16)-19)^ Additionally, when the option was made to use one of these indicators, they were collected in a different manner – without considering the production of all the OHTs or of only the D and OHTn, excluding the OHA – and with other instruments, which makes it impossible to perform comparisons among them.^(20)^

All the variables analyzed after implementation of the OHTs were positively affected by the increase in mean number of professionals employed throughout the years studied. However, some of these results showed a very expressive behavior, such as in the case of the number of supervised tooth brushings (867.62%). This significant increase may have been the result of the combination of a well- structured work process along with the working conditions that exist at these UBSs, where a little more than half offer conditions for the OHTs to carry out clinical procedures concomitant with the other D of the OHTs. This may mean that a large number of these professionals end up directed towards collective activities for much of the time of their work loads, which is very positive, since it is expected that these expressive numbers of supervised brushings and health education might, in a few years, reflect a decrease in the percentage of emergencies in the population of this region, since prevention is being used. The variable of patients seen during the period 2009-2011, showed an expressive growth (91.74%), although lower than that of the other variables analyzed. This variable is directly affected by the number of D that grew, but not in the same proportion as the OHTn and OHA.

It is also possible to observe that almost all the results were most expressive between the years 2009 to 2010 than between 2010 and 2011, which is consistent with the greater growth in the mean number of professionals in the first period mentioned. The exception was the increased number of first dental appointments, likely because there was new orientation from SMS/SP, and the first dental visit began to count the low-risk patients as well (with no history of caries or periodonta alteration) identified in dental triages or during campaigns, indicating a limitation of the present study.

The results for the indicators access, efficiency, and percentage of emergencies during the period analyzed (2009-2011) showed significant changes in favor of the performance of the OHTs. One possible explanation for the increase noted in the indicators access and efficiency might be the previously mentioned growth in number of oral healthcare professionals with a resulting higher offer of services, along with the consolidated work process and interaction that these OHTs acquired over the years.

The monthly analysis of efficiency for the period studied demonstrates an inconsistent behavior in the first months post-implementation, with less expressive variations as of June 2009. For the global period, the indicator showed a slight tendency to increase. The first months of verification of this indicator might reflect the adjustments that were necessary for the implementation and consolidation of the processes established within the scope of action of the OHTs and for which the team originally hired was trained in the month prior to data collection. This effort and motivation in the initial months may have been crucial for the introduction of new professionals and the increase of the population seen to maintain a good performance in efficiency, which resulted in the smaller oscillation observed in the following months.

During the global period, the indicator percentage of emergencies showed a drop, possibly as a reflex of two primary hypotheses: greater understanding of the population as to the work process of the OHTs and therefore, the generation of a small spontaneous demand; or in a reduction of demand of this region repressed throughout the period studies. For the same period (2009-2011), the indicator percentage of absences showed no statistically significant differences, which might reflect that the assigned population still had not changed its posture relative to missing a scheduled appointment. The monthly analysis for these percentage indicators showed a behavior similar to that already described for the efficiency indicator. The plausible explanation for the pattern observed is the same as that offered for efficiency, as to the implementation of the processes and maturing of the OHTs.

We note that the three indictors analyzed monthly (efficiency, percentage of absences and emergencies) demonstrated an abrupt drop in the month of May 2009. We believe such a drop might have been the result of the demands relative to early detection of oral cancer (which occurs along with the influenza vaccination campaign in the municipality of São Paulo), that directs a large part of the OHTs or all of it towards the preventive oral cancer examination in 100% of the elderly who are vaccinated at the UBSs where these OHTs practice. May of 2009 was the first month in which the professionals participated in this campaign, having to concomitantly put forth effort to maintain the implementation of the work processes of the OHTs. Such a scenario was not repeated in the following years, since the professionals, ever more familiar with the work processes, were better prepared and organized for the demands that come up during the campaign period.

When we made joint comparisons of the three statistically significant indicators (access, efficiency, and percentage of emergencies), we observed a few interesting facts that corroborate what has already been explained in other analyses made.

The access indicator showed significant differences in all the annual comparisons, demonstrating once again that the introduction of more professionals and maturing of the work process contributed in an important manner for the access of the population to their first dental appointment. SMS/SP had the goal established for the years 2009, 2010, and 2011 of an access of 3.1%, 4.33%, and 5.0%, respectively, whereas the OHTs of this partnership reached, in all the years, values superior to those hoped for: 5.5% in 2009, 6.5% in 2010, and 9.0% in 2011. The goal for access of the SMS/SP is calculated annually by the same SMS/SP, taking into consideration the municipal population and the result obtained in the previous year by all its OHTs, whether or not a part of the Family Health Strategy.

On the other hand, the significant difference for the efficiency indicator is seen in the comparison between 2009 and 2011, showing that the greatest impact of this indicator happened between the initial and final years of the study, when the OHTs have all the work process well outlined. This indicator also shows that the OHTs of the partnership are within the expected production by SMS/SP (value 4 and 6 for the OHTs modality I and II, respectively). In fact, the lowest mean efficiency was that obtained in 2009 (5.28), when, during the most part of the year, there was only OHTs modality I installed. The efficiency of the OHTs in all the years remained above the values expected by SMS/SP, which can be considered a satisfactory performance. Finally, the significant difference for the percentage of emergencies indicator was observed in the comparison between the years 2009 and 2010. The explanation is the same offered above for variable efficiency.

Additionally, for all the indicators studied, it is important to consider that the maturing of the work process, which happened along the years, was not only among the components of the OHTs, but also among the various healthcare professionals who work in each one of the UBSs, positively impacting the results obtained. Over time, this alignment happened horizontally, enabling a better understanding of the role of healthcare professionals in oral health within the logic of the Family Health Strategy by all in the UBSs, guaranteeing the offer of services in a more organized and coherent way and being capable of adequately absorbing the demand that exists. The community healthcare agent who experienced this insertion intensely should be highlighted, as he/she was trained annually and learned how to deal with all the changes that occurred throughout these years, playing the important role of link between the community and the OHTs.

It also suggests that new projects should be carried out for follow-up of these data along the following years, and that these indicators be compared to the other SMS/SP partners, who work under the same conditions and with the same goals.

## CONCLUSION

We conclude that the data presented in this study express the commitment of these Oral Healthcare Teams, by means of compliance with the work process suggested by the guiding document of the Municipal Health Department of the State of São Paulo, having observed a quantitative improvement in the indicators of efficiency, access, percentage of absences and emergencies for the teams of the partnership between the *Sociedade Beneficente Israelita Brasileira Albert Einstein* and the *Secretaria Municipal de Saúde de São Paulo* during the period of 2009 to 2011.

The results reached along with the goals agreed upon with the Municipal Health Department of the State of São Paulo certainly reflect the increase in number of professionals, the maturing of the work processes of these Oral Healthcare Teams, and of the adequate use of the manual labor available to carry out these activities, besides the bond between these teams with the population and with the other professionals who work at the Primary Care Units.
