# Supplementary material for: Evolution of post-deployment indicators of oral health on the Family Health Strategy
Source: Einstein (Sao Paulo). 2014 Jul-Sep;12(3):274–81. doi: 10.1590/S1679-45082014AO3000 (PMC4872935; doi:10.1590/S1679-45082014AO3000)
Supplement: Translation

# Translation: Evolução de indicadores pós-implantação da saúde bucal na Estratégia Saúde da Família

**Authors:** Danielle da Costa Palacio, Fabiana de Lima Vazquez, Danielle Viana Ribeiro Ramos, Stela Verzinhasse Peres, Antonio Carlos Pereira, Luciane Miranda Guerra, Karine Laura Cortellazzi, Jaqueline Vilela Bulgareli

**Affiliations:** 1 Sociedade Beneficente Israelita Brasileira Albert Einstein, São Paulo, SP, Brasil.; 2 Faculdade de Odontologia de Piracicaba, Piracicaba, SP, Brasil.; 3 Universidade de São Paulo, São Paulo, SP, Brasil.

**Keywords:** Saúde bucal, Saúde da família, Absenteísmo, Acesso aos serviços de saúde, Assistência à saúde

## Abstract

**Objetivo:**

Avaliar a evolução de indicadores após a implantação de 21 Equipes de Saúde Bucal na Estratégia Saúde da Família.

**Métodos:**

Foram utilizados os dados de produção ambulatorial dos profissionais das Equipes de Saúde Bucal, o qual é o instrumento oficial utilizado pela Secretaria Municipal de Saúde de São Paulo para acompanhar o desempenho dos profissionais da saúde bucal em relação a rendimento, acesso, percentual de faltas e de urgências. Esses profissionais trabalharam na parceria entre a Sociedade Beneficente Israelita Brasileira Hospital Albert Einstein e a Secretaria Municipal de Saúde de São Paulo, no período de 2009 a 2011.

**Resultados:**

Para os indicadores: percentual de urgência, rendimento e acesso, houve diferença significativa entre os anos analisados. Para o indicador percentual de faltas, não houve diferença estatisticamente significante. Quando feita a análise mensal, destaca-se que, no início da implantação do serviço, ocorreu oscilação, podendo indicar que o trabalho foi consolidado ao longo dos meses, sendo capaz de receber novos profissionais e aumento da população atendida. Ao se compararem os indicadores do período com as metas pactuadas com a Secretaria Municipal de Saúde de São Paulo, é possível perceber que as Equipes de Saúde Bucal tiveram bom desempenho.

**Conclusão:**

Os resultados alcançados junto às metas pactuadas certamente refletem o aumento do número de profissionais, o amadurecimento dos processos de trabalho dessas Equipes de Saúde Bucal e a otimização da mão de obra disponível para realização das atividades. O entendimento desses resultados é importante para nortear as ações das Equipes de Saúde Bucal para os anos seguintes e avaliar o cumprimento das metas.

## INTRODUÇÃO

As ações da Estratégia Saúde da Família transformaram a forma de cuidar da saúde no Brasil e vem se consolidando como um importante instrumento na promoção do bem-estar da população, com melhoria da saúde e da qualidade de vida. No decorrer dessa transformação, as Unidades Básicas de Saúde (UBS) passaram a contar com equipes multiprofissionais compostas minimamente por um médico, um enfermeiro, um ou dois auxiliares de enfermagem, e de quatro a seis agentes comunitários. Assim, surgiu o desafio do trabalho em equipe multidisciplinar, com responsabilização sobre um território onde vive certo número de famílias. Essa estratégia trouxe a possibilidade de resgatar os vínculos de compromisso e de corresponsabilidade entre os serviços de saúde, os profissionais e a população. Assim, um dos princípios básicos desse modelo de atenção é o de educar para a saúde, indo além da assistência meramente curativa.^(1)-3)^

Para Baldani et al.,^(4)^ o fato de a odontologia não estar presente na Estratégia desde o início possivelmente acarretou prejuízos no processo de integralização dos diversos profissionais de saúde, assim como pode ter determinado formas variadas no processo de implantação das Equipe de Saúde Bucal (ESB).

De acordo com Emmi e Barroso,^(5)^ incorporar a saúde bucal na Estratégia Saúde da Família não significa necessariamente incluir o cirurgião dentista (CD), o auxiliar de saúde bucal (ASB) e o técnico de saúde bucal (TSB) na equipe mínima constituída por médico, enfermeiro e auxiliar de enfermagem, mas exige articular o trabalho desses profissionais ao dessa ESB. A transdisciplinaridade deve ocorrer para um andamento eficiente e resolutivo do trabalho. O processo de trabalho em saúde bucal deve ser compatível e permeável ao processo de trabalho das demais categorias profissionais que atuam nas UBS, no sentido de resolver a maior parte das demandas dessa comunidade assistida, com base no vínculo, no coletivo e na participação dessa população.

Se as Equipes de Saúde da Família (ESF) e ESB funcionarem adequadamente, elas são capazes de resolver 85% dos problemas de saúde da sua comunidade com atendimento de bom nível, prevenindo doenças, evitando internações desnecessárias e melhorando a qualidade de vida da população.^(6, 7)^

Apesar da Estratégia Saúde da Família se apresentar como um modelo reestruturador, verifica-se que ainda existe reprodução de métodos tradicionais.^(7)^ Na implantação das ESF e ESB, torna-se necessário oferecer atenção especial para a qualificação dos profissionais, visando ao atendimento integral das famílias, mudando não apenas o local de atuação, mas, principalmente, a conduta deles frente aos problemas a serem enfrentados^(8, 9)^.

De acordo com Athayde e Rodrigues,^(10)^ embora muito se fale sobre a importância do trabalho em equipe, essa prática encontra vários desafios a serem superados por todos seus componentes. Um deles é o reconhecimento da diversidade de conhecimentos e habilidades entre os membros da equipe, que devem se complementar para enriquecer o trabalho como um todo. No processo de trabalho em saúde, trabalho em equipe e produção de novas práticas em saúde são questões interdependentes e que se enredam numa trama na qual fica difícil definir o que é causa ou consequência.

Para Farias e Moura et al.,^(11)^ reconhecer que existem obstáculos concretos capazes de comprometer o cumprimento dos objetivos da Estratégia Saúde da Família é reconhecer que existem desafios a serem enfrentados.

Diante do exposto, este trabalho teve como desafio estudar as entregas das ESB da parceira entre Sociedade Beneficente Israelita Brasileira Albert Einstein (SBIBAE) e a Secretaria Municipal de Saúde do Estado de São Paulo (SMS/SP), nesse contexto de Estratégia Saúde da Família e no território com múltiplas nuances sociais e populacionais.

## OBJETIVO

Avaliar a evolução dos indicadores de rendimento, acesso, percentual de faltas e percentual de urgências, após a implantação de Equipes de Saúde Bucal na Estratégia Saúde da Família.

## MÉTODOS

No município de São Paulo, a ESF teve início, em 1996, por meio do programa estadual Projeto Qualidade Integral em Saúde (Projeto QUALIS).^(12)^ Esse programa estabeleceu convênios com organizações sem fins lucrativos, que incorporaram recursos assistenciais de apoio às ESF, tais como ESB, saúde mental e ambulatórios de especialidades, antes mesmo da saúde bucal fazer parte oficialmente da Estratégia Saúde da Família.^(13, 14)^

A parceria entre a SBIBAE e a SMS/SP, firmada em 2001, inicialmente só contemplava as ESF na região do Campo Limpo/Vila Andrade, que fica na zona sul do município de São Paulo (SP). Essa região tem população e complexidade equivalentes a de uma cidade de médio porte (mais de 100 mil habitantes), com diversos problemas sociais e muitos contrastes, com regiões de aglomerados populacionais e conjuntos habitacionais de baixa renda, além de condomínios horizontais de média a alta renda.

Embora o termo “Unidade Básica de Saúde” normalmente não seja utilizado em território nacional para tratar de unidades com ESF, aqui foi adotado desse modo, pois é o termo utilizado no Município de São Paulo. Em novembro de 2008, houve a chegada da saúde bucal nessa parceira, com a instalação 12 ESB modalidade I (1 CD e 1 ASB), implantadas em 6 UBS.

As análises das variáveis deste estudo se iniciam a partir de janeiro de 2009. Em abril de 2009, houve o acréscimo de uma ESB modalidade I (total: 13 ESB em 6 UBS). Ainda em 2009, no mês de outubro, mais 5 ESB foram implantadas e essa ampliação passou a atingir 8 UBS (total: 18 ESB em 8 UBS). Outro fato importante foi que 14 ESB da modalidade I (das 18 ESB existentes) passaram para modalidade II (incorporando-se o TSB). Em março de 2011, houve a inclusão de mais uma UBS e a entrada de mais 1 ESB modalidade I e 2 ESB modalidade II (total: 21 ESB em 9 UBS).

O processo de trabalho da ESB esteve baseado nas diretrizes constantes no documento norteador da Prefeitura Municipal de São Paulo – versão 2009,^(15)^ que dispõe de diversos parâmetros, entre os quais 40 horas semanais de trabalho, sendo 30 horas direcionadas ao atendimento clínico, 2 horas às visitas domiciliares, 2 horas às reuniões, 2 horas aos grupos e 4 horas às ações coletivas em escolas da área de abrangência. Devem estar contemplados semanalmente os agendamentos de 49 pacientes para ESB modalidade I e 56 pacientes para ESB modalidade II, bem como atendimento das urgências em demanda espontânea. Além disso, 49 e 56 novos pacientes (primeira consulta odontológica) devem ser contemplados mensalmente para as ESB modalidades I e II, respectivamente, além de percentual de urgências e faltas menores que 20%.

O estudo foi submetido e aprovado pelo Comitê de Ética em Pesquisa da Faculdade de Odontologia de Piracicaba da Universidade Estadual de Campinas (FOP-UNICAMP), sob o protocolo 036/2013, de acordo com a exigência da Resolução 196/96 de pesquisa envolvendo seres humanos, cadastrado por meio da Plataforma Brasil.

A amostra contemplou as UBS onde atuavam as ESB da parceria SBIBAE e SMS/SP na região do Campo Limpo/Vila Andrade, abrangendo toda a população cadastrada no período de 2009 a 2011 no Sistema de Informação da Atenção Básica (SIAB).

Foram utilizados os dados condensados dos mapas de produção ambulatorial dos profissionais das ESB e avaliados os seguintes dados: número total de procedimentos realizados pelos CD, TSB e ASB; número de pacientes agendados; número de consultas não agendadas feitas em caráter de urgência; número de faltas em consultas agendadas; número de atendimentos realizados (pacientes agendados + pacientes de urgência – pacientes que faltaram às consultas); número de casos novos no ano (primeira consulta odontológica); e número total de escovações supervisionadas em ações coletivas (escovações dentais com fins de prevenção, orientadas por um profissional de saúde bucal e em espaço escolar, previamente cadastrado).

Foram então calculados os seguintes indicadores: percentual de faltas (proporção de faltas do total de consultas agendadas), percentual de urgências (proporção de urgências do total de consultas), rendimento (número de procedimentos realizados/número no total de pacientes atendidos) e acesso (número de casos novos /número da população adstrita).

Foi realizada a análise descritiva dos dados por meio de frequências absolutas e relativas, medianas, valores mínimos e máximos. Foi realizado o teste de normalidade para verificar a aderência das variáveis analisadas à curva normal pelo teste de Kolmogorov-Smirnov.

Como as variáveis não apresentaram distribuição normal, optou-se pelos testes livres de distribuição. Para verificar a evolução mensal dos indicadores percentual de faltas, percentual de urgências e rendimento, utilizou-se o teste estatístico não paramétrico de correlação de Spearman. A diferença entre os anos analisados para o indicador de acesso e os demais citados acima foi medida pelo teste não paramétrico de Kruskal-Wallis. Para as análises *post hoc*, utilizou-se o teste de Dunn.

Assumiu-se um nível de 5% para a significância estatística. Os dados foram digitados em uma planilha Excel e analisados pelos programas *Statistical Package for the Social Sciences *(SPSS) versão 20.0 para *Window*s e *GraphPad InStat.*

## RESULTADOS

De acordo com o SIAB, a população cadastrada nas áreas atuantes das ESB, compreendeu à cobertura populacional de 136.749, 167.594 e 182.801 habitantes respectivamente para os anos de 2009, 2010 e 2011, levando-se em consideração o número de UBS com ESB para cada um dos anos citados. Essa população foi formada, em sua maioria, por mulheres (52,8%). Destaca-se que a faixa etária mais predominante, para todos os períodos, foi de 20 a 39 anos (38,3%), seguida dos 40 a 49 anos (12,9%).

A tabela 1 mostra a somatória das atividades das ESB realizadas nos anos de 2009, 2010 e 2011. A tabela 2 mostra o crescimento percentual de 2009 para 2010, de 2010 para 2011, e de 2009 para 2011, das variáveis que foram apresentadas na tabela 1. Para melhor entendimento, ambas as tabelas 1 e 2 são analisadas conjuntamente. Ressalta-se o aumento no número médio de profissionais atuantes durante o período total estudado (crescimento de 78,12%), sendo que em 2010, em relação a 2009, esse acréscimo foi de 56,25% e, em 2011, em relação a 2010, de apenas 14%. De 2009 a 2011, houve um aumento de 128,09% no total de procedimento realizados pelos profissionais integrantes da ESB. De fato, em 2010, observou-se o aumento de 87,35% em relação a 2009, enquanto que, em 2011, em relação a 2010, o aumento foi mais modesto (21,72%). Para o total de atendimentos realizados (pacientes agendados + pacientes de urgências – pacientes faltantes em consultas agendadas), foi observado o aumento de 91,74%, de 2009 a 2011. O aumento observado foi mais expressivo em 2010 (50,28%), em relação a 2009, e consideravelmente menor em 2011 (27,58%), em relação a 2010.

**Tabela 1:** Número absoluto das variáveis analisadas após a implantação das Equipes de Saúde Bucal da região do Campo Limpo/Vila Andrade, em São Paulo, nos anos de 2009, 2010 e 2011

Variável	Ano		
	2009	2010	2011
Média de profissionais atuantes (CD + TSB + ASB)	32	50	57
Total de procedimentos (CD + TSB + ASB)	94.215	176.515	214.896
Total de atendimentos realizados ([PA+PU]-PF)	19.499	29.305	37.389
PA	18.675	30.710	39.457
PU	4.878	6.249	7.204
PF	4.054	7.654	9.272
Pacientes de primeira consulta odontológica	7.588	10.870	16.383
Escovação supervisionada em ação coletiva	7.309	41.569	70.724

CD: cirurgião-dentista; TSB: técnico de saúde bucal; ASB: auxiliar de saúde bucal; PA: pacientes agendados; PU: pacientes de urgência; PF: pacientes faltantes em consultas agendadas.

**Tabela 2:** Comparação percentual entre os anos das variáveis analisadas na tabela 1

Variável	Crescimento (%)		
	2009-2010	2010-2011	2009-2011
Média de profissionais atuantes (CD+TSB+ASB)	56,25	14	78,12
Total de procedimentos (CD+TSB+ASB)	87,35	21,72	128,09
Total de atendimentos realizados ([PA+PU]-PF)	50,28	27,58	91,74
PA	64,44	28,54	111,28
PU	28,10	15,28	47,68
PF	88,80	21,13	128,71
Pacientes de primeira consulta odontológica	43,25	50,71	115,51
Escovação supervisionada em ação coletiva	468,73	70,13	867,62

CD: cirurgião-dentista; TSB: técnico de saúde bucal; ASB: auxiliar de saúde bucal; PA: pacientes agendados; PU pacientes de urgência; PF: pacientes faltantes em consultas agendadas.

Em relação às primeiras consultas odontológicas, observou-se um aumento de 115,51%, de 2009 a 2011. A diferença de 2009 para 2010 e de 2010 para 2011 não foi expressiva (43,25% e 50,71%, respectivamente). Finalmente, constatou-se evolução significativa do número de escovações supervisionadas em escolares, visto que o aumento foi de 867,62% de 2009 a 2011. Esse aumento foi consideravelmente maior no período de 2010 em relação a 2009, em que o crescimento foi de 468,73%, do que em 2011 em relação a 2010, quando o crescimento foi de 70,13%. Os dados foram coletados mensalmente e somados para atingir o total referente a cada ano. O total de procedimentos incorporou os dados para todos os participantes de cada categoria de CD, TSB e ASB.

A tabela 3 mostra os resultados anuais obtidos quanto aos indicadores acesso, rendimento, percentual de faltas e urgências, também ano a ano, para o período 2009-2011. Observa-se que houve uma diferença significativa entre os anos analisados para os indicadores percentual de urgência (p=0,002), rendimento (p=0,009) e acesso (p<0,001). Para o indicador percentual de faltas, não houve diferença estatisticamente significante. Em 2010, constatou-se a menor amplitude entre mínimo e máximo do indicador percentual de faltas entre os anos estudados. O mesmo comportamento foi observado para o indicador percentual de urgências, sendo que o ano de 2010 apresentou a menor disparidade entre mínimo e máximo entre os anos analisados. Deve-se notar uma tendência clara de queda entre os anos de 2009 (mediana de 26,86) e 2011 (mediana de 19,5). A diferença entre o rendimento mínimo e máximo apresentado em 2009 foi a mais díspar entre os anos estudados. O indicador de acesso foi avaliado de forma acumulativa por ano, ou seja, nessa variável, foi feita uma somatória mês a mês para se chegar ao indicador anual. O resultado para o ano de 2011 foi o mais expressivo no período analisado, havendo clara tendência de melhora para tal indicador. Optou-se pelo cálculo da mediana, e não pelo da média, devido à diferença díspar entre mínimo e máximo dos indicadores estudados.

**Tabela 3:** Análise descritiva dos indicadores percentual de faltas, urgências, rendimento e acesso para o período 2009-2011

Variável	2009			2010			2011			Valor de p*
	Mediana	Mínimo	Máximo	Mediana	Mínimo	Máximo	Mediana	Mínimo	Máximo	
Percentual de faltas	22,68	9,3	29,5	25,29	20,1	30,1	23,94	16,5	27,9	0,169
Percentual de urgências	26,86	14,6	36,1	21,52	17,5	24,9	19,5	15,1	22,9	0,002
Rendimento	5,28	2,27	6,21	5,94	4,87	7,14	5,77	4,85	6,81	0,009
Acesso^* *^	5,5			6,5			9,0			<0,001

* Kruskal-Wallis; **o indicador acesso foi avaliado de forma acumulativa por ano; nessa variável está especificado o valor total. Mínimo e máximo referem-se ao menor e maior resultados apresentados entre os meses estudados no período 2009-2011, respectivamente.

Na tabela 4, comparamos os resultados dos indicadores: percentual de faltas, urgências e rendimento para o período 2009-2011, por meio da análise de correlação de Spearman. Observou-se que não houve correlação estatisticamente significativa para o indicador percentual de faltas no período. Em relação ao percentual de urgências, verificou-se correlação negativa significativa. Destaca-se que o indicador rendimento mostrou correlação significativa positiva (r=0,40; p=0,016).

**Tabela 4:** Análise entre os meses de janeiro de 2009 a dezembro de 2011 e para os indicadores percentual de faltas, urgências e rendimentos

Variável	R	Valor de p*
Percentual de faltas	0,18	0,285
Percentual de urgências	-0,58	<0,001
Rendimento médio mensal	0,40	0,016

* Correlação de Spearman.

Para melhor entender o comportamento dos indicadores percentual de faltas, urgências e rendimento médio, de forma que fizessem sentido os dados de correlação encontrados na tabela 3, investigou-se a evolução mensal desses indicadores. O indicador percentual de faltas demonstrou comportamento inconstante nos primeiros meses pós-implantação das ESB, ajustando-se a variações menos expressivas a partir de junho de 2009 (Figura 1). O mesmo comportamento inconstante foi notado nos primeiros meses pós-implantação para o indicador percentual de urgência. As variações observadas para esse indicador, entre os meses no decorrer do tempo de estudo, foram menos expressivas, porém o padrão de distribuição geral denotou ligeira tendência de queda entre os pontos de coleta de dados iniciais e finais. Observou-se redução abrupta de ambos indicadores no mês de maio de 2009, sendo que, no caso do percentual de urgências, a queda foi ainda mais expressiva, principalmente levando em conta dados iniciais coletados para esse indicador.

**Figura 1:** Evolução do percentual de faltas e de urgências, ao longo dos meses, no período 2009-2011

O indicador rendimento (Figura 2) demonstrou um comportamento parecido com os indicadores da figura 1, sendo inconstante nos primeiros meses pós-implantação das ESB e ajustando-se a variações menos expressivas a partir de junho de 2009. O indicador rendimento também demonstrou variações menos expressivas entre os meses seguintes do estudo, com padrão de distribuição geral denotando ligeira tendência de aumento entre os pontos de coleta de dados iniciais e finais. O mês de maio de 2009 também demonstrou uma redução abrupta desse indicador.

**Figura 2:** Evolução do rendimento médio, ao longo dos meses, no período 2009-2011

Destaca-se, na tabela 5, que houve uma diferença estatisticamente significativa entre os anos analisados para os indicadores percentual de urgência (p=0,002), rendimento médio mensal (p=0,009) e acesso (p<0,001). Após a análise de diferenças entre os anos, foi realizada a análise *post hoc* para verificar se a significância estatística se mantinha entre todos os anos. Para o percentual de urgências, verificou-se que houve diferença estatisticamente significativa entre os anos de 2009 e 2010. Quanto ao rendimento médio mensal, a diferença foi observada entre os anos de 2009 e 2011. Em relação ao acesso, a diferença foi verificada para todas as comparações.

**Tabela 5:** *Post hoc* para comparações múltiplas

Comparações	Valor de p*
Percentual de urgências	
2009 *versus* 2010	<0,01
2009 *versus* 2011	>0,05
2010 *versus* 2011	>0,05
Rendimento	
2009 *versus* 2010	>0,05
2009 *versus* 2011	<0,01
2010 *versus* 2011	>0,05
Acesso	
2009 *versus* 2010	<0,01
2009 *versus* 2011	<0,01
2010 *versus* 2011	<0,01

* Teste de Dunn.

## DISCUSSÃO

Neste estudo, apresentamos a evolução dos resultados pós-implantação das 21 ESB da parceria entre SBIBAE e SMS/SP, no período de 2009 a 2011, para uma população geral estimada de 264.697 habitantes, considerando o último ano do estudo. O entendimento desses resultados é importante para nortear as ações de saúde bucal das ESB para os anos seguintes e para avaliar o cumprimento das metas e desafios lançados pela SMS/SP.

Dentre os poucos trabalhos já publicados^(13, 14, 16)^, este é o primeiro que envolveu as parcerias SMS/SP e outras instituições, e verificou a evolução dos indicadores acesso, rendimento, percentual de faltas e urgências das ESB, após a inserção desta na Estratégia Saúde da Família. Os dados disponíveis, referentes às parcerias firmadas pela SMS/SP, ou aqueles publicados por municípios de outros estados focaram no processo de implantação, na cobertura das áreas com esse serviço ou na satisfação do usuário, sem a utilização dos indicadores aqui apresentados.^(5, 6, 16)-19)^ Ou, ainda, quando se fez a opção de utilizar algum desses indicadores, eles foram coletados de forma diferente – sem considerar a produção de todos da ESB ou de apenas CD e TSB, excluindo-se o ASB – e com outros instrumentos, o que impossibilita comparações entre eles.^(20)^

Todas as variáveis analisadas após a implantação das ESB foram positivamente afetadas pelo aumento do número médio de profissionais empregados ao longo dos anos estudados. Entretanto, alguns desses resultados apresentaram um comportamento muito expressivo, como no caso do número de escovações supervisionadas (867,62%). Esse significativo aumento pode ser resultado da combinação de um processo de trabalho bem estruturado aliado às condições de trabalho existentes nessas UBS, onde pouco mais da metade oferece condições para que o TSB realize procedimentos clínicos concomitantes aos demais CD de sua ESB. Isso pode significar que um grande número desses profissionais acaba direcionado para atividades coletivas em boa parte de suas jornadas de trabalho, o que é bastante positivo, pois se espera que esses números expressivos de escovações supervisionadas e de educação em saúde possam, em alguns anos, refletir em uma diminuição do percentual de urgência da população dessa região, uma vez que a prevenção está sendo bastante utilizada. A variável pacientes atendidos, no período 2009-2011, mostrou crescimento expressivo (91,74%), porém menor do que as demais variáveis analisadas. Essa variável é diretamente afetada pelo numero de CD que cresceu, porém não nas mesmas proporções que os TSB e ASB.

Também é possível observar que quase todos os resultados foram mais expressivos entre o período de 2009 a 2010 do que no período de 2010 a 2011, sendo coerente com o maior crescimento do número médio de profissionais no primeiro período citado. A exceção foi o aumento do número de primeiras consultas odontológicas, provavelmente por que houve uma nova orientação da SMS/SP, sendo que a primeira consulta odontológica passou a contabilizar também os pacientes de baixo risco (sem história de cárie ou alteração periodontal) identificados em triagens odontológicas ou em campanhas, indicando uma limitação do presente estudo.

Os resultados para os indicadores acesso, rendimento e percentual de urgências no período analisado (2009-2011) mostraram mudanças significantes favoráveis ao desempenho da ESB. Uma possível explicação para o aumento observado nos indicadores acesso e rendimento pode ser o já referido crescimento do número de profissionais de saúde bucal com consequente maior oferta dos serviços, aliado ao processo de trabalho consolidado e entrosamento que essas ESB adquiriram ao longo dos anos.

A análise mensal do rendimento para período estudado demonstra um comportamento inconstante nos primeiros meses pós-implantação, tendendo a variações menos expressivas a partir de junho de 2009. Para o período global, o indicador mostrou ligeira tendência de aumento. Os primeiros meses de aferição desse indicador podem refletir os ajustes que se fizeram necessários para a implantação e a consolidação dos processos estabelecidos no escopo de atuação das ESB e para os quais a equipe originalmente contratada foi treinada no mês anterior ao primeiro de coleta de dados. Esse esforço e motivação, nos meses iniciais, podem ter sido cruciais para que a introdução de novos profissionais e o aumento da população atendida mantivessem um bom desempenho do rendimento, o que resultou na menor oscilação observada nos meses seguintes.

No período global, no indicador percentual de urgências, verificou-se uma queda, provavelmente reflexo de duas principais hipóteses: um maior entendimento da população sobre o processo de trabalho das ESB e, portanto, a geração de menor demanda espontânea; ou uma redução da demanda reprimida dessa região ao longo do período estudado. Para o mesmo período (2009-2011), o indicador percentual de faltas não mostrou diferença estaticamente significativa, o que pode refletir que a população adstrita ainda não tenha mudado sua postura em relação ao comparecimento em consulta agendada. A análise mensal para esses indicadores percentuais mostrou comportamento parecido com aquele já descrito para o indicador rendimento. A explicação plausível para o padrão observado é a mesma oferecida para o rendimento, no que tange à implementação dos processos e ao amadurecimento das ESB.

Nota-se que os três indicadores analisados mensalmente (rendimento, percentual de faltas e urgências) demonstraram uma queda brusca no mês de maio de 2009. Acredita-se que tal queda possa ter sido resultado das demandas relativas à campanha de detecção precoce de câncer de bucal (que ocorre junto à campanha de vacinação de influenza no município de São Paulo), a qual direciona grande parte da ESB ou toda ela para o exame preventivo de câncer bucal de 100% dos idosos vacinados nas UBS em que estas ESB atuam. Maio de 2009 foi o primeiro mês em que os profissionais participaram dessa campanha, tendo que concomitantemente se esforçarem para manter a implantação dos processos de trabalho das ESB. Tal cenário não se repetiu nos anos seguintes, pois os profissionais, além de mais familiarizados com os processos de trabalho, estavam melhores preparados e organizados para as demandas que surgem em período de campanha.

Quando fazemos comparações agregadas dos três indicadores estatisticamente significantes (acesso, rendimento e percentual de urgências), observamos alguns fatos interessantes e que corroboram o já exposto em outras análises feitas.

O indicador acesso mostrou diferenças significativas em todas as comparações anuais, mostrando novamente que a introdução de mais profissionais e o amadurecimento do processo de trabalho contribuíram de maneira importante para o acesso da população à primeira consulta odontológica. A SMS/SP tinha como meta estabelecida para os anos de 2009, 2010 e 2011 um acesso de 3,1%, 4,33% e 5,0%, respectivamente, sendo que as ESB dessa parceria atingiram, em todos os anos, valores superiores aos pactuados: 5,5% em 2009, 6,5% em 2010 e 9,0% em 2011. A meta de acesso da SMS/SP é calculada anualmente pela própria SMS/SP, levando-se em consideração a população municipal e o resultado alcançado no ano anterior por todas as suas ESB, sendo ou não parte da Estratégia Saúde da Família.

Já a diferença significante para o indicador rendimento verifica-se na comparação entre os 2009 e 2011, mostrando que o maior impacto desse indicador se deu entre o ano inicial e final do estudo, quando as ESB estão com todo o processo de trabalho bastante alinhado. Esse indicador também mostra que as ESB da parceria estão dentro da produção esperada pela SMS/SP (valor 4 e 6 para as ESB modalidade I e II, respectivamente). De fato o menor rendimento médio foi o obtido em 2009 (5,28), quando, na maior parte do ano, só houve ESB modalidade I instalada. O rendimento das ESB ficou, em todos os anos, acima dos valores esperados pela SMS/SP, o que pode ser considerado um desempenho satisfatório. Finalmente, a diferença significativa para o indicador percentual de urgências foi observada na comparação entre os anos de 2009 e 2010. A explicação é a mesma oferecida acima para a variável rendimento.

Ademais, para todos os indicadores estudados, vale considerar que o amadurecimento do processo de trabalho, que aconteceu ao longo dos anos, foi não somente entre os componentes das ESB, mas também entre os diversos profissionais de saúde que atuam em cada uma das UBS, impactando de forma positiva nos resultados obtidos. Com o passar dos anos, esse alinhamento aconteceu de forma horizontal, possibilitando um melhor entendimento do papel dos profissionais de saúde bucal na lógica da Estratégia Saúde da Família por todos nas UBS, garantindo oferta dos serviços de forma mais organizada e coerente e sendo capaz de absorver de maneira adequada a demanda existente. Destaque deve ser dado ao agente comunitário de saúde que viveu toda essa inserção intensamente, sendo treinado anualmente e aprendendo a lidar com todas as mudanças que ocorreram ao longo desses anos, sendo um importante elo de ligação entre comunidade e ESB.

Sugere-se que novos trabalhos devam ser realizados para o acompanhamento desses dados ao longo dos demais anos e para comparação desses indicadores com os demais parceiros da SMS/SP, que trabalham sob a mesma as mesmas condições e com as mesmas metas.

## CONCLUSÃO

Conclui-se que os dados apresentados neste estudo expressam o comprometimento dessas Equipes de Saúde Bucal, por meio da adesão ao processo de trabalho sugerido pelo documento norteador da Secretaria Municipal de Saúde do Estado de São Paulo, observando-se melhora quantitativa dos indicadores de rendimento, acesso, percentual de faltas e urgências para das equipes da parceria entre a Sociedade Beneficente Israelita Brasileira Albert Einstein e a Secretaria Municipal de Saúde do Estado de São Paulo durante o período de 2009 a 2011.

Os resultados alcançados junto às metas pactuadas com a Secretaria Municipal de Saúde do Estado de São Paulo certamente refletem o aumento do número de profissionais, o amadurecimento dos processos de trabalho dessas Equipes de Saúde Bucal e o adequado uso da mão de obra disponível para realização dessas atividades, além do vínculo dessas equipes com a população e com os demais profissionais que atuam nas Unidades Básicas de Saúde.
